# The Improvement and Related Mechanism of Microecologics on the Sports Performance and Post-Exercise Recovery of Athletes: A Narrative Review

**DOI:** 10.3390/nu16111602

**Published:** 2024-05-24

**Authors:** Keer Yang, Yonglin Chen, Minghan Wang, Yishuo Zhang, Yu Yuan, Haoyang Hou, Yu-Heng Mao

**Affiliations:** 1 School of Exercise and Health, Guangzhou Sport University, Guangzhou 510500, China; keryang014@126.com (K.Y.); chenyl946@163.com (Y.C.); wangmh516@163.com (M.W.); 18736412692@163.com (Y.Z.); yuany@gzsport.edu.cn (Y.Y.); houhaoyang2001@163.com (H.H.); 2 Guangdong Key Laboratory of Human Sports Performance Science, Guangzhou Sport University, Guangzhou 510500, China

**Keywords:** microecologics, sports performance, antifatigue, probiotic, prebiotic, gut microbiota

## Abstract

The diversity and functionality of gut microbiota may play a crucial role in the function of human motor-related systems. In addition to traditional nutritional supplements, there is growing interest in microecologics due to their potential to enhance sports performance and facilitate post-exercise recovery by modulating the gut microecological environment. However, there is a lack of relevant reviews on this topic. This review provides a comprehensive overview of studies investigating the effects of various types of microecologics, such as probiotics, prebiotics, synbiotics, and postbiotics, on enhancing sports performance and facilitating post-exercise recovery by regulating energy metabolism, mitigating oxidative-stress-induced damage, modulating immune responses, and attenuating bone loss. Although further investigations are warranted to elucidate the underlying mechanisms through which microecologics exert their effects. In summary, this study aims to provide scientific evidence for the future development of microecologics in athletics.

## 1. Introduction

Repetitive high-intensity and high-volume training, coupled with intense competition, are commonly observed in athletics. However, insufficient post-exercise recovery can result in a decline in athletes’ physiological functions and performance. Nutritional support stands as one of the crucial factors for athletes to optimize their performance and facilitate post-exercise recovery. In recent decades, researchers have discovered that the diversity and composition of gut microbes directly or indirectly influence athletes’ immunity, energy metabolism, nutrient absorption, and oxidation levels under varying exercise conditions. Consequently, these factors are closely intertwined with sports performance. Therefore, identifying agents capable of modulating gut microbiota has emerged as an effective approach to enhance sports performance.

**Figure 1:**
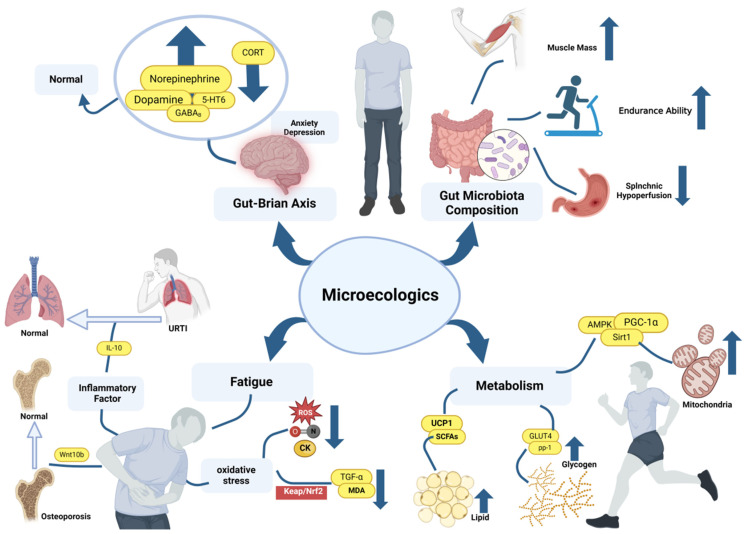
Promotion mechanisms of microecologics on sports performance. GABA_B_: gamma-aminobutyric acid (GABA) is the main inhibitory neurotransmitter of the CNS, GABA_B_ is a receptor in the constituent GABA structure; CORT: corticosterone, excessive corticosterone levels reflect the appearance of anxiety symptoms; 5-HT6: Human 5-hydroxytryptamine receptor 6 belongs to the human serotonin receptor family and plays an important role in regulating emotion and cognition; AMPK/Sirt1/PGC-1α is an important signaling pathway that activates mitochondrial activity; GLUT4: glucose transporters type 4 is important in skeletal muscle glucose uptake for muscle contraction; pp-1: is glycogen synthesis-related genes, promoting glycogen synthesis in the body; UCP1: uncoupling protein 1, adjust heat production in human brown fat to avoid low metabolism or hypothermia; ROS, NO, CK can serve as a physiological indicator of the degree of oxidative stress in vivo or vitro; Keap1/Nrf2: It is closely associated with anti-oxidation, and it reduce the harm of oxidation products to the body by reducing TGF-α expression and malondialdehyde (MDA) production. The blue straight arrow represents a rise or fall in expression.

Microecologics, as a group of substances capable of regulating gut microbiota and subsequently impacting health improvement, have the ability to modulate microbial metabolite production, gastrointestinal physiology, and immune modulation. The market for functional foods and nutritional supplements related to microecologics has been expanding greatly in recent years. The estimated market size for microecologics was about USD 850 billion in 2023, with a significant compound annual growth rate trend. Furthermore, broader research has shown that the effects of microecologics are not limited to chronic intestinal or systematic metabolic diseases but have also garnered increasing attention in recent years for their promising effects on enhancing sports performance in the athletic population [1,2,3,4]. However, there is a scarcity of reviews focusing on the effects and signaling pathways of microecologics specifically within this population. Thus, this review investigates whether microecologics can enhance athletes’ physical and mental performance (Table 1) while further exploring their underlying mechanisms for promoting effects (Figure 1), thus substantiating the positive influence of microecologics in optimizing sports performance and facilitating post-exercise recovery.

This narrative review was performed in Pubmed, MEDLINE, Science Direct and Web of Science using the following key words: exercise, gut microbiota, probiotics, prebiotics, athlete, synbiotics, postbiotics, metabolism, immune, bone, muscle and oxidative stress. Articles published from 1 January 1994 to 1 May 2024 were included. By conducting searches using these key words or their synonyms, we reviewed the titles and abstracts of all manuscripts. Exclusion criteria were applied if: (i) the study data fell outside of the proposed timeline; (ii) the topics were not relevant to the focused purpose of the study; or (iii) they were not in English. All authors conducted searches for relevant manuscripts for summary discussion and co-writing.

## 2. Microecologics

Based on recent epidemiological and physiological studies, a significant portion of human health and disease may be influenced by microbial communities. These microbiota consist of bacteria, fungi, viruses, and other organisms, with the majority inhabiting the intestinal system. These microorganisms play a crucial role in shaping the diversity and composition of gut microbiota, which in turn affect the host’s digestive, metabolic, and immune antioxidant abilities. The typical microecologics include probiotics, prebiotics, synbiotics and postbiotics [22,23]. Numerous studies have reported therapeutic effects of microecologics on chronic diseases through restoration of gastrointestinal function [24,25], immunity [26], improvement in energy utilization and overall metabolism [27]. However, research on how microecologics can enhance human exercise performance is still at an early stage. In this regard, microecologics are categorized into four types based on their biological activity and composition while exploring their impact on sports performance.

### 2.1. Probiotics

The Food and Agriculture Organization (FAO) and the World Health Organization (WHO) have defined probiotics as “living microorganisms that, when ingested in sufficient quantity, can confer health benefits to the host” [28]. In other words, probiotics are a type of microorganism that can benefit the host by altering the composition of specific parts of the body’s microbiota [29]. The majority of microbiota in the gastrointestinal tract is concentrated in the colon where it acts as a natural bioreactor fermenting and utilizing substances from upper GI tract while producing metabolites such as short-chain fatty acids (SCFAs) and secondary bile acids [30]. Currently, probiotics have been reported to directly stimulate the host’s metabolic and immune systems [31,32]. Probiotics enhance the absorption and utilization of glycogen by increasing the activity of GLUT4, AMPK and Akt [33]. This may be an effective way to mediate athletic glucose imbalance after acute exercise. Additionally, their metabolites can serve as a source of energy, such as SCFAs, to ensure that the host can exercise for longer periods [34]. In terms of immune systems, probiotics can improve immunomodulatory mechanisms by stimulating intestinal immune cell activity such as B cells [35]. Based on the physiological role of probiotics in the host, several studies have explored the sports performance-enhancing effects of probiotics. For instance, a 6-week oral administration of *Lactobacillus plantarum* TWK10 improved aerobic capacity in healthy adults [36], and mice orally treated with *Lactobacillus plantarum* PL-02 for 4 weeks (a human-origin probiotic derived from a weightlifting gold medalist) exhibited reduced levels of excess metabolites and improvements in muscle mass, strength, and endurance performance [7]. The combination of exercise and supplementation with *Bifidobacterium longum subsp. Longum* OLP-01 for a duration of 6 weeks in mice not only enhanced endurance and grip strength, but also mitigated inflammation and injury markers [9]. Some other studies have reported differences in the fecal microbial composition between athletes and sedentary individuals, revealing that athletes exhibit a higher α-diversity of gut microbiota, indicating an enrichment of bacterial richness [29]. Additionally, certain studies have demonstrated a correlation between *Akkermansia* sp. and *Prevotella* spp. with high metabolic levels [37]. Furthermore, the quantity of SCFAs in athletes’ feces also increased, thereby enhancing host’s immunity and metabolism. In a study of marathon runners, Scheiman et al. found that the genes related to the metabolism of lactic acid and propionic acid in fecal microbiota increased after Marathon. Then, the selected strains were isolated from feces and administered to mice, resulting in a 13% improvement in treadmill running time compared to the control group [38]. However, there remains a dearth of research investigating the impact of probiotics derived from elite athletes’ feces on human subjects [29]. To date, most intervention studies focusing on probiotics in athletes have predominantly centered around lactic acid bacteria and bifidobacteria [39].

### 2.2. Prebiotics

Prebiotics refer to a group of organic substances that are indigestible and non-absorbable by the host, but can selectively promote the metabolism and proliferation of host microorganisms conferring health benefits to the host [40,41]. The main types of prebiotics include functional oligosaccharides, polysaccharides, certain protein hydrolysates, and natural plant extracts [42]. Due to their tolerance to acidic environments in the gastrointestinal tract, prebiotics are not easily digested by the small intestine and can smoothly reach the colon for utilization through bacterial fermentation, so it can selectively stimulate the composition changed and enhance the metabolism of gut microbiota and human health [43,44]. Moreover, prebiotic can decrease the rate of postprandial blood glucose increased, improve the body’s immunity, and reduce the accumulation of inflammatory factors in the body [45]. Functional oligosaccharides and polysaccharides have been widely utilized as nutritional supplements for athletes. They are known to alleviate glycogen depletion and protect the intestinal barrier function [46]. Additionally, protein hydrolysates are primarily aimed at regulating skeletal muscle function [47]. More recently, a significant number of studies have demonstrated that flavonoids and polyphenols extracted from natural plants can enhance the host’s energy level by modulating the intestinal barrier function and gut microbial composition [48,49]. The increasing research on a wider range of prebiotics provides athletes with more options to meet their specific dietary patterns in their preferred ways.

### 2.3. Synbiotics

Synbiotics are defined as a combination of live microorganisms and substrate(s) that have the potential to confer health benefits on the host according to the latest definition from the ISAPP in 2019 [50]. The ISAPP indicated that synbiotics can be classified into two subcategories. Complementary synbiotics contain probiotics and prebiotics that target autochthonous microorganisms, while synergistic synbiotics contain substrates that are utilizable by the co-administered microorganisms. So far, these two categories have not been separately investigated in systematic reviews [51]. The presence of this complex can compensate for the insufficiency of probiotic intake alone, enhance the survival rate of probiotics in the gastrointestinal tract, modulate the microecological composition of the host intestine, and subsequently confer advantageous effects on the host [52]. A study investigated the synergistic effect of synbiotics in improving immune function compared to using probiotics or prebiotics individually. This supplementation including 200 mg Glycomax Immunoglobulin, 50 mg Glycomax Lactoferrin, and 100 mg CBAR-Blend-100 contained 4.6 × 10^8^ *L. casei* 431, 6 × 10^8^ BB-12, 4.6 × 10^8^ *Lactobacillus acidophilus* LA-5, 4.6 × 10^8^ *Lactobacillus rhamnosus* GG, 90 mg Raftiline, 10 mg Raftilose GR and 10 mg magnesium stearate. Following a 21-day monitoring period involving 22 physically healthy males undergoing training, it was observed that supplementation with synbiotics significantly reduced serum IL-10 concentration compared to all single intervention groups. This finding suggests a stronger immune function associated with synbiotics in athletes [17]. 

### 2.4. Postbiotics

The definition of postbiotics was proposed by the international Scientific Association for Probiotics and Prebiotics (ISAPP) in 2021 to be “a preparation of inanimate microorganisms and/or their components that confers a health benefit on the host” [53]. In other words, postbiotics encompass not only complete inactivated microorganisms (excluding live microorganisms) but also structural fragments of microorganisms. With the continuous deepening of research, metabolic and secreted products produced by microbes during growth and fermentation, such as SCFAs, secondary bile acids, exopolysaccharides (EPS), and other substances can also be classified as postbiotics [54,55]. For instance, the administration of heat-killed *Lactiplantibacillus plantarum* TWK10 to healthy adults for 6 weeks significantly enhanced sports performance, reduced fatigue-related features, and increased muscle mass proportion [5]. Supplementing acetate and succinate enhanced mitochondrial respiratory function and increased mitochondrial content in mouse skeletal muscle [56].

## 3. Microecologics Improve the Function of Locomotor System and Physical Performance

### 3.1. The Promotive Effects on Bone System

The bone is an organ that is generated through a dynamic balance between osteoblasts and osteoclasts [57]. Early studies have demonstrated the significance of gut microbiota in bone metabolism, with disruptions in gut function leading to loss of bone mass [58]. Through the gut–bone axis, the gut microbiota exerts regulatory effects on bone metabolism by modulating host metabolism, immune regulation, hormone secretion, and other physiological processes. The targeted modulation of gut microbiota using microecologics supplementation can effectively mitigate bone mass loss or enhance bone mineral density (BMD) [59,60]. This effect is advantageous in alleviating bone loss or osteoporosis following high-intensity exercise. Two studies have demonstrated that *Lactobacillus reuteri* can effectively prevent the onset of osteoporosis by inhibiting bone trabeculae loss and enhancing bone density [11,61]. Additionally, several common prebiotics such as Galactooligosaccharides (GOS) and xylo-oligosaccharide (XOS) have been reported to augment intestinal absorption of calcium and magnesium ions, stimulate osteoblast activity, and suppress osteoclast activity through probiotic growth stimulation [13,14]. A study on soluble corn fiber and calcium absorption found that healthy adolescent females could increase their calcium absorption after four weeks of supplementation through stimulating the fermentation and utilization of gut microbiota [62]. In order to further explore the preventive and therapeutic effects of microecologics on bone loss, Lucas [63] treated mice with propionate and butyrate. The results showed that these SCFAs were associated with the inhibition of osteoclast differentiation and bone resorption. These collective effects provide a strong scientific basis for microecologics to promote bone health in athletes. Consequently, microecologics hold great promise as strategies for preventing post-exercise bone loss resulting from heavy workloads or excessive training.

### 3.2. Improvement of Skeletal Muscle Content and Strength by Microecologics

Skeletal muscle, the largest organ in the human body, serves as a fundamental regulator of energy metabolism and homeostasis, while also playing a pivotal role in movement. The mass of skeletal muscle reflects bodily growth and development, nutritional equilibrium, and physical performance to a certain extent. In addition to diet and exercise, the intestinal microecosystem significantly influences nutrient absorption and utilization, consequently impacting host body composition. Several studies have demonstrated that germ-free (GF) mice exhibit decreased muscle mass along with reduced expression of key factors involved in bone–muscle metabolism such as succinate dehydrogenase and peroxisome proliferator-activated receptor γ coactivator 1α (PGC-1α) [64].

Based on the gut–muscle axis concept identified, this confirms that both gut microbes and their derived metabolites can act on muscle metabolism; recent progress indicates that modulation of the expression level of the gut–muscle axis by nutrient supplementation is beneficial for the stabilization and elevation of muscle metabolism [65]. Several investigations focusing on age-related muscle mass loss in mice have documented that microecologics effectively mitigate muscle lost and suppress the release of inflammatory factors by regulating gastrointestinal function [66]. With the further expansion of research, scientists have developed an intervention combining exercise and microecologics to validate the interconnection between motor function and gut microbiota [67]. For instance, supplementation with *Lactobacillus plantarum* PL-02 combined with resistance training can enhance muscle mass and exercise performance while reducing fatigue-inducing byproducts [6]. The results of another study investigating the effects of 6 weeks of *Lactobacillus plantarum* TWK10 supplementation revealed significant improvements in muscle mass and handgrip strength compared to the placebo group [68]. The metabolites produced by these probiotics, such as SCFAs, secondary bile acids, and certain amino acids, have the potential to enhance muscle mass and strength [18,69]. A study conducted on 26-month-old mice with age-related muscle loss demonstrated that supplementation of butyrate effectively attenuated muscle atrophy by increasing the cross-sectional area of muscle fibers, accompanied by enhanced glucose metabolism and elevated mitochondrial content in skeletal muscle [70]. Additionally, a study involving healthy menopausal women revealed that butyrate influenced muscle mass [24]. However, current research and treatments primarily focus on chronic diseases, leaving a dearth of studies investigating whether SCFAs exert similar effects in healthy adults or athletes. Regarding postbiotics, other studies have shown that oral intake of heat-killed *Bifidobacterium breve* B-3 (HKB-3) resulted in increased grip strength after 2 or 4 weeks and improved distribution of oxidative fibers in the gastrocnemius muscle, ultimately promoting muscle hypertrophy and modification [19].

### 3.3. The Promotive Effects of Microecologics on Muscular Endurance

Muscular endurance is defined as the ability of muscle group(s) to perform repeated contractions over a specific period of time until causing fatigue [71]. The availability of adequate oxygen and energy substrates is one of the major factors determining muscular endurance capacity. So far, many studies have consistently demonstrated the potential of probiotics to enhance endurance performance in both animal models and human subject [7,72]. After administering *Lactobacillus plantarum* PS128 as a supplement to triathletes for a duration of four weeks, it was observed that the relative abundance of pathogenic bacteria decreased, while the proportion of beneficial bacteria increased. Additionally, there was an elevation in SCFAs concentration in fecal samples. Notably, compared to the placebo group, the endurance capacity also exhibited significant improvement [73]. The supplementation of *Lactobacillus plantarum* PS128 for 8 weeks in another study on triathletes demonstrated a significant reduction in creatine kinase (CK) content. Additionally, the release of intense exercise-induced inflammatory factors and oxidative stress markers was also observed to be diminished [8]. The gut microbiota has been demonstrated to impact endurance performance in mice through the production of SCFAs [20]. Additionally, another study revealed that acetic acid treatment led to increased phosphorylation of AMPK in both liver and skeletal muscle cells. The utilization of glucose and fatty acids in L6 cells was enhanced with acetic acid treatment [74]. Microecologics can also demonstrate anti-fatigue activity by mitigating excessive formation of oxidants induced by high-intensity training or alleviate symptoms such as upper respiratory tract infections (URTI) and gastrointestinal (GI) symptoms that arise during exhaustive exercise, thereby resulting in diminished exercise performance [15,21,75]. Several studies have demonstrated that probiotics can ameliorate oxidative stress and muscle damage following long-distance running [76]. Additionally, they have been shown to reduce the incidence of immune diseases through the modulation of salivary immunoglobulin A (SIgA) levels and a decrease in inflammatory factors within the body. Similarly, a 12-week supplementation of synbiotics containing *Lactobacillus acidophilus* CUL-60 and CUL-21, *Bifidobacterium bifidum* CUL-20, *Bifidobacterium animalis subspecies lactis* CUL-34, and fructooligosaccharides among novice long-distance triathletes resulted in improved gastrointestinal permeability, thereby maximizing the reduction in gastrointestinal injury symptoms [77]. Conversely, some findings suggest that probiotic supplementation may not alleviate infection symptom severity [10,78,79], possibly due to variations in athletes’ body composition and metabolism leading to individual differences in the efficacy of probiotics.

## 4. Improvement of Mental Performance by Microecologics

Anxiety, depression, and other mental disorders often manifest in athletes as they prepare for important competitions. These negative emotions can lead to the development of various gastrointestinal complications through the gut–brain axis [80]. Therefore, it is crucial for athletes to address and alleviate gut disorders and excessive negative emotions. For instance, a study demonstrated that a 6-week supplementation of probiotics resulted in reduced anxiety- and depression-related behaviors among badminton players. This effect could potentially be attributed to the modulation of gut microbiota, leading to decreased corticosterone secretion and increased norepinephrine secretion [81]. Another study conducted on individuals with limited diversity in their gut microbiota demonstrated that supplementation of *Bifidobacterium animalis* subsp. *lactis* BB-12 improved emotional states and increased the abundance of bifidobacteria. However, it should be noted that the assessment of anxiety levels in this experiment relied solely on subjective cognition of the participants, and objective physiological data reflecting anxiety were not collected [12]. Importantly, both studies only monitored participants’ dietary intake without implementing any changes to their dietary patterns.

## 5. Promotion Mechanisms of Microecologics on Exercise Ability

### 5.1. Improvement of Gut Microbial Composition

Previous studies have demonstrated a substantial disparity in gut microbiota diversity between athletes or physically active adults and sedentary individuals. Carlo Bressa et al. reported significant differences in the fecal microbiome of physically active females compared to their sedentary counterparts, specifically highlighting variations in 11 genera including *Faecalibacterium prausnitzii* and *Akkermansia muciniphila* [82]. A certain quantity of microecologics can enhance the host’s composition. In a study, *Lactobacillus. plantarum* PL-02 was isolated from the feces of an Olympic female weightlifting gold medalist and utilized as a dietary supplement for mice. The results demonstrated that supplementation with *L. plantarum* PL-02 significantly increased both *L. plantarum* and *Akkermansia muciniphila* richness in the gut microbiota of mice after 4 weeks, leading to significant improvements in endurance performance, muscle mass, muscle strength, and other indices [7]. In another study, administration of *Lactobacillus reuteri* to antibiotic-treated mice with intestinal disturbances resulted in a reduction in the ratio of Firmicutes to Bacteroidetes fecal ratio and alleviated bone loss associated with intestinal disorders [11].

In recent years, prebiotics have garnered increasing attention due to their remarkable impact on gut microbiota, as well as their high safety profile, broad applicability, and ample resources. Certain prebiotics, particularly functional oligosaccharides and polysaccharides, have been shown to enhance physical performance by modifying the composition of gut microbes. A study conducted by Camilla J Williams et al. investigated the synergistic effect of FOS-enriched inulin combined with high-intensity interval training (HIIT) in 40 sedentary yet evidently healthy adults. The findings revealed that the incorporation of FOS-enriched inulin led to a modulation of gut microbial composition, potentially associated with an enhanced ventilatory threshold. However, no statistically significant increase in VO_2_ peak was observed when compared to the intervention involving HIIT alone [83]. Furthermore, a separate study demonstrated that β-glucan supplementation increased the abundance of *Bifidobacterium* and *Lactobacillus* in fecal samples, while also improving handgrip strength among athletes and reducing plasma concentrations of inflammatory markers after exercise, indicating a lower risk of tissue injury [84,85]. Similarly, mice supplemented with Neoagarotetraose can resist intensive exercise injury by altering the β-diversity of gut microbiota composition, which indicated the potential anti-fatigue effect of Neoagaroteraose [86]. There may be varying degrees of physiological effects in the supplementation of prebiotics in different states; most prebiotics serve as a long-term nutritional supplement to improve the body’s metabolic function, but there are few studies as a short-term transient nutritional tonic [87]. And the most commonly used prebiotic supplements belong to oligosaccharides, a carbohydrate, and we all know that athletes supplemented with carbohydrates during exercise can prolong the time to fatigue [88], but whether this acute transient supplementary intake also regulates the composition of the gut microbiota requires some further findings.

Plasma citrulline is currently acknowledged as a biomarker reflecting intestinal function, wherein a dysbiosis model of gut microbiota associated with plasma citrulline demonstrates a decline in the abundance of Streptococcaceae and Lachnospiraceae [89]. Moreover, a study demonstrated the prebiotic effect of citrulline and highlighted the synergistic contribution of *Lactobacillus helveticus* strain to enhance intestinal epithelial barrier function [90]. Another study showed that pre-exercise oral citrulline can effectively mitigate splanchnic hypoperfusion resulting from excessive physical exertion, a condition associated with gastrointestinal injury, thereby preventing the decline in exercise performance phenomenon [91].

### 5.2. Improving Mental State through Microbiota–Gut–Brain Axis

Emerging evidence highlights the role of gut microbiota in facilitating bidirectional signaling between the gut and the brain, suggesting that these microbial communities establish a connection between the enteric nervous system (ENS) and central nervous system (CNS) via the autonomic nervous system (ANS), known as the microbiota–gut–brain axis. On one hand, bioactive molecules released by ENS can exert influence on brain function, particularly in regulating emotions and neural reflexes [92]. On the other hand, the CNS is able to regulate activities such as GI peristalsis and digestive juices secretion. Some studies report that anxiety and depression are associated with intestinal dysbiosis, which can be attributed to the bidirectional communication within the gut–brain axis [93,94]. Enhanced clinical evidence increasingly suggests that probiotics possess the capacity to mitigate chronic fatigue and ameliorate symptoms of depression and anxiety by modulating neurotransmitter and hormone metabolism. An animal study showed that long-term supplementation with *Lactobacillus rhamnosus JB-1* reduced stress-induced corticosterone levels, as well as associated behaviors such as anxiety and depression. Furthermore, the expression of GABA_B_ receptors was augmented in the JB-1 group, which plays a pivotal role in symptomatology related to anxiety or depressive disorders [95]. Several studies have also demonstrated that *Lactobacillus plantarum*-EVs (*L*-EVs) and *L. plantarum* IS-10506 can effectively reverse the decreased expression of brain-derived neurotrophic factor (BDNF) in the hippocampus of mice, while *L*-EVs have shown potential in alleviating symptoms of depression and anxiety induced by glucocorticoids [96,97]. Moreover, the administration of a probiotic strain *Lactobacillus plantarum* DR7 has been demonstrated to upregulate the expression levels of neurotransmitters such as 5-HT6 and TPH2. These neurotransmitters play crucial roles in stress adaptation, and their low levels have been correlated with an increased risk of anxiety and depression. Furthermore, this probiotic intervention effectively alleviated symptoms of stress and anxiety in the subjects [97]. According to statistics, about 16–34% of the elite athletes show depression-related symptoms [98]. In the current social background of athletes’ mental states gradually receiving attention, microecologics are accepted by athletes as a new way to improve their mental state. However, the research on human psychological state is not as objective as in animals, and may have more complex social environment or partner effects on subjects. Therefore, it is crucial to conduct more comprehensive studies on how microecologics can enhance mental well-being among athletes, particularly for alleviating symptoms of anxiety and depression.

### 5.3. Enhancing the Activity of AMPK

AMP-activated protein kinase (AMPK) is a key sensor in the regulation of biological energy metabolism, being activated by various conditions such as hypoxia, ischemia, exercise, and other stimuli [99]. Previous studies have demonstrated that the biological activity of AMPK is triggered when the AMP:ATP or ADP:ATP ratios decrease due to energy consumption [100], so that it stimulates glucose uptake and lipid oxidation in skeletal muscle, thus enhancing the contractility of skeletal muscle [101]. Moreover, AMPK can also mediate the AMPK/PGC-1α pathway to regulate mitochondrial biogenesis, providing the necessary energy substrate for ATP synthesis. Many studies have shown that the gut microecosystem can participate in body energy synthesis and utilization via regulating various signaling pathways mediated by AMPK [19,102,103]. In an animal experiment, the administration of COSs for 6 weeks significantly increased the expression levels of Sirt1 and AMPK in rat muscle, both of which play important roles in mitochondrial biosynthesis. The results indicate that COSs intervention can effectively enhance mitochondrial biosynthesis by regulating the activity of PGC-1α via AMPK and Sirt1 signaling pathways [104]. In another animal study, supplementation of HKB-3 was found to activate AMPK activity in response to a decrease in the ATP/AMP ratio. Furthermore, it enhanced mitochondrial biogenesis content through the AMPK-PGC-1α signaling pathway, thereby providing energy substrates for mitochondrial energy metabolism [19]. Furthermore, the study also investigated the impact of HKB-3 on muscle types in rats and observed that HKB-3 administration led to an elevation in Akt regulatory factor levels in soleus muscle through the mTOR signaling pathway. Consequently, this resulted in increased skeletal muscle mass and a higher content of oxidized muscle fibers in the gastrocnemius muscle of rats [104]. Unexpectedly, the results showed that both of the level of AMPK and mTOR are increased with HKB-3 supplementation, which conflicted with most studies, indicating that the activity of AMPK increases alongside inhibition of mTOR expression in vivo. The authors postulated that this discrepancy may be due to the complex composition of HKB-3, including lipoteichoic acid, peptidoglycan, nucleotides and some other components, and each substance may participate in the AMPK-PGC-1α signaling pathway. To further elucidate the key responsible components within HKB-3, additional investigations should focus on studying its major functional constituents individually. The experiment included both B-3 and HKB-3 as intervention, and both appeared to induce the production of mitochondrial organisms within skeletal muscle. Of note, HKB-3 improved skeletal muscle-related functions more than B-3; it may be that HKB-3 is better absorbed by the host to stimulate the metabolism of the gut microbiota in a better quality and mild pathway [105]. This result has high significance in the use of postbiotics as a nutritional supplement.

### 5.4. Improving Glycogen and Lipid Metabolism

Carbohydrates and lipids are the predominant fuel sources during exercise and physical activities, particularly in endurance exercises above moderate intensity. Numerous studies have demonstrated a positive correlation between the abundance of SCFAs and high-level sports performance [106]. Additionally, SCFAs have been established as energy substrates involved in the body’s energy metabolism, thereby augmenting exercise performance and physiological functions [107]. Kim’s study group found that supplementation with *Bifidobacterium lactis* HY8101 improved diabetes-induced plasma total cholesterol and triglyceride (TG) levels, while also increasing muscle glycogen content. Furthermore, in the skeletal muscle tissue of mice treated with HY8101, the expression of GLUT 4 was upregulated. These findings suggest that HY8101 enhances body function in diabetic mice by improving glucose metabolism and lipid metabolism in tissues [108]. Subsequently, an experiment investigating the eight-week supplementation of *Bifidobacterium animalis lactis* BL-99 (BL-99) revealed elevated levels of acetate, butyrate, and propionate. Correlation analysis indicated a significant positive association between bifidobacteria abundance and SCFAs as well as polyunsaturated fatty acids (PUFAs), which have been shown to enhance fat oxidation. Additionally, SCFAs contribute to stabilizing blood glucose levels and promoting glycogen metabolism. Consequently, BL-99 supplementation contributes to improved host energy metabolism, resulting in increased muscle strength and VO_2max_ [109]. A study reported that Chitooligosaccharides (COSs) alleviated lipid and glucose metabolism disorders in obese mice. Experimental findings demonstrated that COSs upregulated the expression of UCP1 in subcutaneous adipose tissue, a major thermogenic factor responsible for subcutaneous fat thermogenesis. Ultimately, this led to a decreased body fat ratio in the mice [110].

### 5.5. Reduce the Damage Caused by Oxidative Stress

Athletes usually undergo intensive or exhaustive exercise that easily leads to the excessive accumulation of oxygen free radicals, which leads to high oxidative stress characterized by DNA hydroxylation, protein denaturation and cell apoptosis within the human body [111]. Excessive levels of oxygen free radicals are believed to accelerate or exacerbate fatigue [112] and negatively affect sports performance. In recent years, many studies have found that microecologics alleviate fatigue after intensive or exhaustive exercise through scavenging free radicals, inhibiting excessive oxidation reaction and enhancing antioxidant enzyme activity, among other mechanisms. Previous studies have proved that heat-killed postbiotics can reduce the activity of CK and LDH produced by high-intensity exercise and delay the generation of fatigue in athletes [21].

A study reported that a 6-week experimental intervention involving the supplementation of *Lactobacillus paracei* PS23 in general adults aged 20–40 years resulted in a significant improvement in the antioxidant capacity of the human body, as well as a reduction in exercise-induced muscle damage (EIMD), including muscle injury and inflammation [113]. Compared with the placebo group, the experimental group exhibited significant reductions in serum concentrations of CK and thiobarbituric acid reactive substances (TBARS) at 24 and 48 h, as well as a significant decrease in myoglobin levels at 48 h. These findings collectively suggest that *L. paracei* PS23 can mitigate exercise-induced muscle damage by attenuating oxidative stress. The same research team also conducted a further analysis to investigate the mechanisms by which *L. paracei* PS23 reduces oxidation. They found that mitochondrial function decreased in aged mice after supplementation with *L. paracei* PS23, while the levels of SOD and GPx were enhanced in the *L. paracei* PS23 group [114]. Additionally, Zhang observed an increase in running time among groups with elevated antioxidant enzyme activity, indicating that supplementation with microecologics helps to improve the anti-fatigue ability of athletes [115].

Not only do probiotics exhibit effects against oxidative stress, but their metabolites also demonstrate similar properties. Studies have revealed that acetate and butyrate enhance mitochondrial respiration in mice under conditions of oxidative stress, with butyrate primarily inhibiting the production of reactive oxygen species (ROS) and acetate mainly suppressing nitric oxide (NO) production [116]. Polysaccharides such as *Lycium barbarum* polysaccharide (LBP), Ganoderma lucidum polysaccharide (GL-PS), and *Radix pseudostellariae* polysaccharides (RPPs) have been shown in several studies to enhance the activity levels of superoxide dismutase (SOD), glutathione peroxidase (GPX), and catalase (CAT) [110,117,118,119]. However, further regulated signaling pathway validation about LBP, GL-PS, and RPPS is still lacking. For examples, one of these studies provided in-depth evidence that LBP effectively mitigated oxidative stress-induced injury by modulating the Keap1/Nrf2 antioxidative stress signaling pathway in rats subjected to vigorous exercise. In comparison to the exhaustive exercise (EX) group, supplementation with LBP for 5 weeks resulted in decreased expression of Keap1 and increased expression of Nrf2, pNrf2, GCLM, and GCLC in the EX + LBP group [118]. Keap1/Nrf2 signaling pathway, as a critical regulatory node in antioxidant and anti-aging, has gradually found to be a crucial determinant in many disease phenotypes [120], while LBP as a potential prebiotic shows great antioxidant potential, and it provides a scientific basis for microecologics to relieve the oxidative stress caused by excessive exercise in athletes.

### 5.6. Regulating on the Immune System

High-volume training is commonly employed by endurance athletes and often results in a decline in immune function when insufficient post-exercise recovery is provided [121]. Chronic fatigue leads to the accumulation of inflammatory factors in the human body, which disrupts immune system regulation and increases susceptibility to pathogenic infections, particularly upper respiratory tract infections (URTIs). URTIs triggered by long-term exercise training in athletes are accompanied by fatigue, limiting training capacity and impairing performance. Additionally, this condition is associated with decreased levels of salivary secretory immunoglobulin A (SIgA) as well as reduced counts of T lymphocytes, B cells, natural killer (NK) cells, and neutrophils [122]. Additionally, the therapeutic effect of a multi-strain probiotic containing *Lactobacillus acidophilus* CUL-60, *Lactobacillus acidophilus* CUL-21, *Bifidobacterium bifidum* CUL-20, and *Bifidobacterium animalis subsp lactis* CUL-34 on upper respiratory tract infections (URTI) in marathon runners was investigated. The results demonstrated that the administration of multi-strain probiotics significantly reduced the incidence of URTI and enhanced interleukin-10 (IL-10) production in monocytes [123]. In fact, numerous probiotic strains have been found to possess potent immunomodulatory properties, capable of augmenting macrophage activity and regulating immunoglobulin secretion to enhance host immune function. Additionally, they can restore GI microecosystem homeostasis by bolstering the secretion of the GI epithelial barrier and mucus, thereby promoting overall gastrointestinal health [32]. The administration of Hsp65-producing *Lactococcus lactis* in a murine model demonstrated a preventive effect on arthritis onset, while concurrently enhancing the activity of T-cells and B-cells [124]. Due to the temporary reduction in immune function caused by intense exercise, there is a decrease in the body’s TNF-α content, leading to an increased susceptibility to microbial infections. Therefore, TNF-α can be used as an indicator to evaluate the host immune function. Another study observed that the intake of *Sparassis crispa* for 8 weeks resulted in higher levels of TNF-α in mouse plasma compared with the mice with vigorous exercise. Additionally, there was an increase in MyD88 expression, which is a canonical adaptor for inflammatory signaling pathways downstream of Toll-like receptor (TLR) and interleukin-1 (IL-1) receptor families [124]. 

### 5.7. Relieving Bone Loss Caused by Excessive Glucocorticoid

Glucocorticoids, as metabolic hormones, play a pivotal role in metabolism. During high-intensity anaerobic exercise or prolonged aerobic exercise, there is a significant elevation in the levels of glucocorticoids, especially the main hormone cortisol [125]. Many previous studies have concluded that negative stress and excessive training intensity can lead to the accumulation of cortisol [126]. Despite being widely used as an immunomodulatory drug in clinical settings [127], glucocorticoids can cause muscle atrophy, osteoporosis, and other diseases when present in abnormally high concentrations. Glucocorticoid-induced osteoporosis is characterized by a reduction in bone formation [128]. A study demonstrated that excessive glucocorticoid administration induced alterations in the gut microbiota composition in mice and suppressed Wnt10b protein expression through the Wnt/β-catenin signaling pathway, which plays a crucial role in embryonic development, tissue integrity, and stem cell activity [129]. Consequently, this led to decreased osteoblast activity, increased osteoclast activity, and inhibition of osteocyte proliferation, ultimately triggering osteoporosis. Conversely, supplementation with *Lactobacillus reuteri* was found to attenuate the upregulation of apoptotic factors such as BAX/BCL-2 in GC-Tx-treated 16-week-old male mice and alleviate the suppression of Wnt10b protein expression [130]. Another study on *Lactobacillus reuteri* ATCC 6475 (*L. reuteri* 6457) also demonstrated that supplementation for 8 weeks relieved gut barrier dysfunction and trabecular bone loss induced by glucocorticoids in male mice [131]. In conclusion, these findings provide evidence that oral microecologics can prevent glucocorticoid-induced bone cell loss. This may be a good nutritional supplement to maintain bone system health for athletes, which work in high-intensity exercise or under excessive psychological stress. But the *L. reuteri* study is about a male mice model of glucocorticoid induction, and it can only demonstrate that *L. reuteri* supplementation alleviates the suppression of osteoblasts after vigorous exercise, but not for pre-exercise prophylaxis, and there are a lack of findings about female mice. The team then examined whether supplementation with *L. reuteri* could increase bone mineral density in female mice and found that only the inflammatory female mice were improved cortical bone parameters [132]. Therefore, more studies are needed to confirm whether *L. reuteri* is resistant to bone loss in female mice or athletes. But the bone metabolism always needs a dynamic balance between osteoblasts and osteoclasts, and excessive osteoblast expression will lead to high bone mineral density, which is not conducive to maintain the stability of bone metabolism [133]. Therefore, more research is needed to confirm that microecologics are a class of dynamically regulated substances, which are not self-defeating by excessive activation of osteoblast activity. Furthermore, further research about how *L. reuteri* reduces glucocorticoid release to resist bone loss is also needed.

## 6. Conclusions

Targeted intake of nutritional supplements has a positive impact on athletes, alleviating post-exercise fatigue and enhancing sports performance. Scientists have gained a comprehensive understanding of the gut microbiota, leading to recognition in the sports community that optimizing the composition and function of human gut microbiota can potentially improve exercise performance. Supplementation with microecologics such as probiotics, prebiotics, synbiotics, and postbiotics facilitates functional enhancement of gut microbiota, regulating host energy metabolism, immune response modulation, oxidative stress levels, and other aspects. In-depth research on various kinds of microecologics shows their broad development prospects in the field of sports as potential applications for athletes undergoing intensive training. They may store and restore more energy for the body during training or competition, similar to how marathon runners replenish electrolytes and water during races. However, several limitations still persist in the existing studies. Firstly, there is a need for more comprehensive research involving modern isolation and functional verification techniques to explore novel microecologics from diverse sources, including fermented foods, traditional Chinese medicine, algae, and other marine resources. Secondly, due to the unique nature of the athletic population, there is still limited evidence regarding the beneficial effects of microecologics in athletes, although some animal experiments have shown promising results. Therefore, more high-quality human experiments with larger sample sizes are urgently needed to conclusively determine the impact of microecologics on sports performance and post-exercise recovery. Thirdly, individual differences should be considered when studying microecologics in athletes as well as other populations; precision nutrition is essential to enhance sports performance. In summary, urgent attention is required towards conducting further studies on enhancing sports performance through microecologics while delving deeper into the biochemical mechanisms involved among athletes and physically active individuals to improve both competitive outcomes and public health.

## Figures and Tables

**Table 1:** Promoting effect of different types of microecologics on exercise performance.

Supplements	Reference	Doses	Duration	Subjects	Sample, Size	Pathways	Main Findings
**Probiotic**							
*L. plantarum* TWK10	[5]	0, 2.05 × 10⁸, or 1.03 × 10⁹ CFU/kg/d	6 weeks	Mice	n = 24 (all male)	Reduce inflammation through gut-muscle axis	Increased muscle mass, improved forelimb grip strength
*L*. *plantarum* PL-02	[6]	2.05 × 10^9^ CFU/kg/d	4 weeks	Mice	n = 32 (all male)	Generate SCFAs to improve the activity of AMPK and GLUT4	
	[7]	0, 2.05 × 10^9^, 4.10 × 10^9^ and 1.03 × 10^10^ CFU/kg/d	4 weeks	Mice	n = 40 (all male)	/	Reduce excess metabolic products
*L*. *Plantarum* PS128	[8]	Two capsules (3 × 10^10^ CFU/capsule)	4 weeks	Human	n = 8 (male 4, female 4)	Improve microbial composition	Improve endurance, lower limb explosive strength, lower muscle damage indices et.al
*Lactobacillus casei* Shirota	[9]	3 × 10^10^ (CFU) (80 mL/bottle)	6 weeks	Human	n = 30 (not mentioned)	Gut–brain axis	Reduce competitive anxiety, perceived stress
	[10]	6.5 × 10^9^ live LcS/pot	16 weeks	Human	n = 84 (male 54, female 30)	Increase SIgA content	Reduce the incidence of URTI
*Lactobacillus reuteri*	[11]	3.3 × 10^8^ CFU/mL	4 weeks	Mice	n = 30 (all male)	Improve microbial composition	Increase the quantity and activity of osteoblasts
*Bifidobacterium animalis* subsp. *lactis* BB-12	[12]	1 × 10^9^ CFU/100 g	8 weeks	Human	n = 21 (all male)	Increase the abundance of *Bifidobacteriaceae*	Improve cognitive state anxiety, somatic state anxiety, and anxiety emotion
**Prebiotic**							
GOSs	[13]	0, 2, 4, 6, or 8% GOS by weight	8 weeks	Mice	n = 75 (all male)	Improve microbial composition	Increase the quantity and activity of osteoblasts, increase the gut absorption of Ca^2+^ and Mg^2+^
XOSs	[14]	0, 1, 2, or 4% by concentration	30 days	Mice	n = 96 (all male)	Upregulate the expression of (TRPV6) and Na^+^/Ca^2+^ transporters	Increase BMD
Glutamine	[15]	0.9 g/kg of fat-free mass per day	7 days	Human	n = 8 (male 5, female 3)	Activate HSF-1	Reduce increased GI permeability and intestinal cellular damage
Konjac glucomannan	[16]	1.25, 2.50, and 5.00 mg/mL	42 days	Mice	n = 30 (all male)	Increase the abundance of bacteroidetes	Increase tolerance to excessive exercise
**Synbiotic**							
Biosource^TM^ Gut Balance, Probiotech Pharma, Chr. Hansen A/S, Horsholm, Denmark	[17]	(Described in 2.3)	21 days	Human	n = 22 (all male)	Improve gut microbial composition	Elicit a 14-fold increase in the recovery of fecal *L. paracasei.*
**Postbiotic**							
Butyrate	[18]	high-fat diet at 5% *wt/wt*	10 weeks	Mice	/	Increase Mitochondrial Biogenesis	Increase muscle mass
HKB-3	[19]	1 × 10^9^ CFU/rat	4 weeks	Rats	n = 52 (all male)		
Acetate	[20]	1 μL/h	6 weeks	Mice	n = 12 (all male)	As an energy source for glycogen synthesis	Improve endurance ability
*HKL.lactis* JCM 5805	[21]	100 billion cells of HK LC-Plasma per day	13 days	Human	n = 25 (all male)	Activate pDC activity	Alleviate the symptom of URTI and fatigue caused by high intensity

## Abbreviations

FAO, Food and Agriculture Organization; WHO, World Health Organization; SCFAs, short chain fatty acids; EPS, exopolysaccharides; BMD, bone mineral density; GOS, galactooligosaccharides; XOS, xylo-oligosaccharide; URTI, upper respiratory tract infections; SIgA, salivary immunoglobulin A; GI, gastrointestinal; HIIT, high-intensity interval training; ENS, enteric nervous system; CNS, central nervous system; ANS, autonomic nervous system; GABA_B,_ AMPK, AMP-activated protein kinase; PGC-1α, peroxisome proliferator-activated receptor γ coactivator 1α; COSss, Chitooligosaccharides; GF, germ-free; HKB-3, heat-killed *Bifidobacterium breve* B-3; GLUT 4, glucose transporters type 4; EIMD, exercise-induced muscle damage; CK, creatine kinase; TBARS, thiobarbituric acid reactive substances; EPS, extracellular polysaccharide; PUFAs, polyunsaturated fatty acids.

## References

[1] Zhu H., Ren Z., Zang Y., Hua H., Lu J., Xu Q., Zhu S. (2020). Effects of Microecological Preparations on Obese Patients after Bariatric Surgery: A Systematic Review and Meta-Analysis. Evid.-Based Complement. Altern. Med..

[2] Clauss M., Gérard P., Mosca A., Leclerc M. (2021). Interplay between Exercise and Gut Microbiome in the Context of Human Health and Performance. Front. Nutr..

[3] (2023). Probiotics Market Size, Share & Trends Analysis Report by Product (Food & Beverages, Dietary Supplements), by Ingredient (Bacteria, Yeast), by Distribution Channel, by End-Use, by Region, and Segment Forecasts, 2023–2030. https://www.grandviewresearch.com/industry-analysis/probiotics-market.

[4] (2022). Prebiotics Market Size, Share & Trends Analysis Report by Ingredients (FOS, Inulin, GOS, MOS), by Application (Food & Beverages, Dietary Supplements, Animal Feed), by Region, and Segment Forecasts, 2022–2030. https://www.grandviewresearch.com/industry-analysis/prebiotics-market.

[5] Lee C.C., Liao Y.C., Lee M.C., Cheng Y.C., Chiou S.Y., Lin J.S., Huang C.C., Watanabe K. (2022). Different Impacts of Heat-Killed and Viable *Lactiplantibacillus plantarum* TWK10 on Exercise Performance, Fatigue, Body Composition, and Gut Microbiota in Humans. Microorganisms.

[6] Yeh W.L., Hsu Y.J., Ho C.S., Ho H.H., Kuo Y.W., Tsai S.Y., Huang C.C., Lee M.C. (2022). *Lactobacillus plantarum* PL-02 Supplementation Combined with Resistance Training Improved Muscle Mass, Force, and Exercise Performance in Mice. Front. Nutr..

[7] Lee M.C., Hsu Y.J., Ho H.H., Kuo Y.W., Lin W.Y., Tsai S.Y., Chen W.L., Lin C.L., Huang C.C. (2021). Effectiveness of Human-origin *Lactobacillus plantarum* PL-02 in Improving Muscle Mass, Exercise Performance and Anti-fatigue. Sci. Rep..

[8] Meng X., Gao Y., Qi H., Ding Y., Sun Y. (2022). Clinical Application Value of *Lactobacillus Plantarum* PS128 in Patients with Anxiety Disorders. Clin. Psychopharmacol. Neurosci..

[9] Aykut M.N., Erdoğan E.N., Çelik M.N., Gürbüz M. (2024). An Updated View of the Effect of Probiotic Supplement on Sports Performance: A Detailed Review. Curr. Nutr. Rep..

[10] Gleeson M., Bishop N.C., Oliveira M., Tauler P. (2011). Daily Probiotic’s (*Lactobacillus casei* Shirota) Reduction of Infection Incidence in Athletes. Int. J. Sport Nutr. Exerc. Metab..

[11] Schepper J.D., Collins F.L., Rios-Arce N.D., Raehtz S., Schaefer L., Gardinier J.D., Britton R.A., Parameswaran N., McCabe L.R. (2019). Probiotic *Lactobacillus reuteri* Prevents Postantibiotic Bone Loss by Reducing Intestinal Dysbiosis and Preventing Barrier Disruption. J. Bone Min. Res..

[12] Dong W., Wang Y., Liao S., Tang W., Peng L., Song G. (2020). *Bifidobacterium animalis* subsp. lactis BB-12 Improves the State Anxiety and Sports Performance of Young Divers Under Stress Situations: A Single-Arm, Prospective Proof-of-Concept Study. Front. Psychol..

[13] Weaver C.M., Martin B.R., Nakatsu C.H., Armstrong A.P., Clavijo A., McCabe L.D., McCabe G.P., Duignan S., Schoterman M.H., van den Heuvel E.G. (2011). Galactooligosaccharides Improve Mineral Absorption and Bone Properties in Growing Rats through Gut Fermentation. J. Agric. Food Chem..

[14] de Sire A., de Sire R., Curci C., Castiglione F., Wahli W. (2022). Role of Dietary Supplements and Probiotics in Modulating Microbiota and Bone Health: The Gut-Bone Axis. Cells.

[15] Zuhl M.N., Lanphere K.R., Kravitz L., Mermier C.M., Schneider S., Dokladny K., Moseley P.L. (2014). Effects of Oral Glutamine Supplementation on Exercise-Induced Gastrointestinal Permeability and Tight Junction Protein Expression. J. Appl. Physiol..

[16] Mao Y.H., Wang M., Yuan Y., Yan J.K., Peng Y., Xu G., Weng X. (2023). Konjac Glucomannan Counteracted the Side Effects of Excessive Exercise on Gut Microbiome, Endurance, and Strength in an Overtraining Mice Model. Nutrients.

[17] West N.P., Pyne D.B., Cripps A.W., Christophersen C.T., Conlon M.A., Fricker P.A. (2012). Gut Balance, A Synbiotic Supplement, Increases Fecal *Lactobacillus Paracasei* but has Little Effect on Immunity in Healthy Physically Active Individuals. Gut Microbes.

[18] Gao Z., Yin J., Zhang J., Ward R.E., Martin R.J., Lefevre M., Cefalu W.T., Ye J. (2009). Butyrate Improves Insulin Sensitivity and Increases Energy Expenditure in Mice. Diabetes.

[19] Wang Y., Li Y., Bo L., Zhou E., Chen Y., Naranmandakh S., Xie W., Ru Q., Chen L., Zhu Z. (2023). Progress of Linking Gut Microbiota and Musculoskeletal Health: Casualty, Mechanisms, and Translational Values. Gut Microbes.

[20] Okamoto T., Morino K., Ugi S., Nakagawa F., Lemecha M., Ida S., Ohashi N., Sato D., Fujita Y., Maegawa H. (2019). Microbiome Potentiates Endurance Exercise through Intestinal Acetate Production. Am. J. Physiol. Endocrinol. Metab..

[21] Komano Y., Shimada K., Naito H., Fukao K., Ishihara Y., Fujii T., Kokubo T., Daida H. (2018). Efficacy of Heat-Killed *Lactococcus lactis* JCM 5805 on Immunity and Fatigue during Consecutive High Intensity Exercise in Male Athletes: A Randomized, Placebo-Controlled, Double-Blinded Trial. J. Int. Soc. Sports Nutr..

[22] Fan Y., Pedersen O. (2021). Gut Microbiota in Human Metabolic Health and Disease. Nat. Rev. Microbiol..

[23] Zhou M., Huang J., Zhou J., Zhi C., Bai Y., Che Q., Cao H., Guo J., Su Z. (2023). Anti-Obesity Effect and Mechanism of Chitooligosaccharides Were Revealed Based on Lipidomics in Diet-Induced Obese Mice. Molecules.

[24] Lv W.Q., Lin X., Shen H., Liu H.M., Qiu X., Li B.Y., Shen W.D., Ge C.L., Lv F.Y., Shen J. (2021). Human Gut Microbiome Impacts Skeletal Muscle Mass via Gut Microbial Synthesis of the Short-chain Fatty Acid Butyrate among Healthy Menopausal Women. J. Cachexia Sarcopenia Muscle.

[25] Ouwehand A.C. (2024). Journal of Functional Foods special issue: Probiotics, prebiotics, microbiota and health. J. Funct. Foods.

[26] Isolauri E., Sütas Y., Kankaanpää P., Arvilommi H., Salminen S. (2001). Probiotics: Effects on Immunity. Am. J. Clin. Nutr..

[27] Hibberd A.A., Yde C.C., Ziegler M.L., Honoré A.H., Saarinen M.T., Lahtinen S., Stahl B., Jensen H.M., Stenman L.K. (2019). Probiotic or Synbiotic Alters the Gut Microbiota and Metabolism in a Randomised Controlled Trial of Weight Management in Overweight Adults. Benef. Microbes.

[28] Hill C., Guarner F., Reid G., Gibson G.R., Merenstein D.J., Pot B., Morelli L., Canani R.B., Flint H.J., Salminen S. (2014). Expert Consensus Document. The International Scientific Association for Probiotics and Prebiotics Consensus Statement on the Scope and Appropriate Use of the Term Probiotic. Nat. Rev. Gastroenterol. Hepatol..

[29] Yoo S., Jung S.C., Kwak K., Kim J.S. (2024). The Role of Prebiotics in Modulating Gut Microbiota: Implications for Human Health. Int. J. Mol. Sci..

[30] Oniszczuk A., Oniszczuk T., Gancarz M., Szymańska J. (2021). Role of Gut Microbiota, Probiotics and Prebiotics in the Cardiovascular Diseases. Molecules.

[31] Kim S.K., Guevarra R.B., Kim Y.T., Kwon J., Kim H., Cho J.H., Kim H.B., Lee J.H. (2019). Role of Probiotics in Human Gut Microbiome-Associated Diseases. J. Microbiol. Biotechnol..

[32] La Fata G., Weber P., Mohajeri M.H. (2018). Probiotics and the Gut Immune System: Indirect Regulation. Probiotics Antimicrob. Proteins.

[33] Kim Y.A., Keogh J.B., Clifton P.M. (2018). Probiotics, Prebiotics, Synbiotics and Insulin Sensitivity. Nutr. Res. Rev..

[34] Green M., Arora K., Prakash S. (2020). Microbial Medicine: Prebiotic and Probiotic Functional Foods to Target Obesity and Metabolic Syndrome. Int. J. Mol. Sci..

[35] Mazziotta C., Tognon M., Martini F., Torreggiani E., Rotondo J.C. (2023). Probiotics Mechanism of Action on Immune Cells and Beneficial Effects on Human Health. Cells.

[36] Huang W.C., Lee M.C., Lee C.C., Ng K.S., Hsu Y.J., Tsai T.Y., Young S.L., Lin J.S., Huang C.C. (2019). Effect of *Lactobacillus plantarum* TWK10 on Exercise Physiological Adaptation, Performance, and Body Composition in Healthy Humans. Nutrients.

[37] Cani P.D., de Vos W.M. (2017). Next-Generation Beneficial Microbes: The Case of *Akkermansia muciniphila*. Front. Microbiol..

[38] Scheiman J., Luber J.M., Chavkin T.A., MacDonald T., Tung A., Pham L.D., Wibowo M.C., Wurth R.C., Punthambaker S., Tierney B.T. (2019). Meta-omics Analysis of Elite Athletes Identifies a Performance-Enhancing Microbe that Functions via Lactate Metabolism. Nat. Med..

[39] Cox A.J., Pyne D.B., Saunders P.U., Fricker P.A. (2010). Oral Administration of the Probiotic *Lactobacillus fermentum* VRI-003 and Mucosal Immunity in Endurance Athletes. Br. J. Sports Med..

[40] Yadav M.K., Kumari I., Singh B., Sharma K.K., Tiwari S.K. (2022). Probiotics, Prebiotics and Synbiotics: Safe Options for Next-generation Therapeutics. Appl. Microbiol. Biotechnol..

[41] Ashaolu T.J., Ashaolu J.O., Adeyeye S.A.O. (2021). Fermentation of Prebiotics by Human Colonic Microbiota In Vitro and Short-Chain Fatty Acids Production: A Critical Review. J. Appl. Microbiol..

[42] Al-Sheraji S.H., Ismail A., Manap M.Y., Mustafa S., Yusof R.M., Hassan F.A. (2013). Prebiotics as Functional Foods: A review. J. Funct. Foods.

[43] Markowiak P., Śliżewska K. (2017). Effects of Probiotics, Prebiotics, and Synbiotics on Human Health. Nutrients.

[44] Quigley E.M.M. (2019). Prebiotics and Probiotics in Digestive Health. Clin. Gastroenterol. Hepatol..

[45] Li D.D., Ma J.M., Li M.J., Gao L.L., Fan Y.N., Zhang Y.N., Tao X.J., Yang J.J. (2022). Supplementation of *Lycium barbarum* Polysaccharide Combined with Aerobic Exercise Ameliorates High-Fat-Induced Nonalcoholic Steatohepatitis via AMPK/PPARα/PGC-1α Pathway. Nutrients.

[46] Tiller N.B., Roberts J.D., Beasley L., Chapman S., Pinto J.M., Smith L., Wiffin M., Russell M., Sparks S.A., Duckworth L. (2019). International Society of Sports Nutrition Position Stand: Nutritional Considerations for Single-Stage Ultra-Marathon Training and Racing. J. Int. Soc. Sports Nutr..

[47] Morgan P.T., Breen L. (2021). The Role of Protein Hydrolysates for Exercise-Induced Skeletal Muscle Recovery and Adaptation: A Current Perspective. Nutr. Metab..

[48] Wan M.L.Y., Co V.A., El-Nezami H. (2021). Dietary Polyphenol Impact on Gut Health and Microbiota. Crit. Rev. Food Sci. Nutr..

[49] Baky M.H., Elshahed M., Wessjohann L., Farag M.A. (2022). Interactions between Dietary Flavonoids and the Gut Microbiome: A Comprehensive Review. Br. J. Nutr..

[50] Marco M.L., Sanders M.E., Gänzle M., Arrieta M.C., Cotter P.D., De Vuyst L., Hill C., Holzapfel W., Lebeer S., Merenstein D. (2021). The International Scientific Association for Probiotics and Prebiotics (ISAPP) Consensus Statement on Fermented Foods. Nat. Rev. Gastroenterol. Hepatol..

[51] Chan C.K.Y., Tao J., Chan O.S., Li H.B., Pang H. (2020). Preventing Respiratory Tract Infections by Synbiotic Interventions: A Systematic Review and Meta-Analysis of Randomized Controlled Trials. Adv. Nutr..

[52] Krumbeck J.A., Walter J., Hutkins R.W. (2018). Synbiotics for Improved Human Health: Recent Developments, Challenges, and Opportunities. Annu. Rev. Food Sci. Technol..

[53] Vinderola G., Sanders M.E., Salminen S. (2022). The Concept of Postbiotics. Foods.

[54] Scott E., De Paepe K., Van de Wiele T. (2022). Postbiotics and Their Health Modulatory Biomolecules. Biomolecules.

[55] Prajapati N., Patel J., Singh S., Yadav V.K., Joshi C., Patani A., Prajapati D., Sahoo D.K., Patel A. (2023). Postbiotic Production: Harnessing the Power of Microbial Metabolites for Health Applications. Front. Microbiol..

[56] Ismaeel A., Valentino T.R., Burke B., Goh J., Saliu T.P., Albathi F., Owen A., McCarthy J.J., Wen Y. (2023). Acetate and Succinate Benefit Host Muscle Energetics as Exercise-Associated Post-biotics. Physiol. Rep..

[57] Tomaszewska E., Dobrowolski P., Świetlicka I., Muszyński S., Kostro K., Jakubczak A., Taszkun I., Żmuda A., Rycerz K., Blicharski T. (2018). Effects of Maternal Treatment with β-hydroxy-β-metylbutyrate and 2-oxoglutaric Acid on Femur Development in Offspring of Minks of the Standard Dark Brown Type. J. Anim. Physiol. Anim. Nutr..

[58] Quach D., Britton R.A. (2017). Gut Microbiota and Bone Health. Adv. Exp. Med. Biol..

[59] Zaiss M.M., Jones R.M., Schett G., Pacifici R. (2019). The Gut-Bone Axis: How Bacterial Metabolites Bridge the Distance. J. Clin. Investig..

[60] Cheng S., Qi X., Ma M., Zhang L., Cheng B., Liang C., Liu L., Li P., Kafle O.P., Wen Y. (2020). Assessing the Relationship between Gut Microbiota and Bone Mineral Density. Front. Genet..

[61] Malmir H., Ejtahed H.S., Soroush A.R., Mortazavian A.M., Fahimfar N., Ostovar A., Esmaillzadeh A., Larijani B., Hasani-Ranjbar S. (2021). Probiotics as a New Regulator for Bone Health: A Systematic Review and Meta-Analysis. Evid. Based Complement. Altern. Med..

[62] Whisner C.M., Martin B.R., Nakatsu C.H., Story J.A., MacDonald-Clarke C.J., McCabe L.D., McCabe G.P., Weaver C.M. (2016). Soluble Corn Fiber Increases Calcium Absorption Associated with Shifts in the Gut Microbiome: A Randomized Dose-Response Trial in Free-Living Pubertal Females. J. Nutr..

[63] Lucas S., Omata Y., Hofmann J., Böttcher M., Iljazovic A., Sarter K., Albrecht O., Schulz O., Krishnacoumar B., Krönke G. (2018). Short-Chain Fatty Acids Regulate Systemic Bone Mass and Protect from Pathological Bone Loss. Nat. Commun..

[64] Lahiri S., Kim H., Garcia-Perez I., Reza M.M., Martin K.A., Kundu P., Cox L.M., Selkrig J., Posma J.M., Zhang H. (2019). The Gut Microbiota Influences Skeletal Muscle Mass and Function in Mice. Sci. Transl. Med..

[65] Nucci R.A.B., Filho V.A.N., Jacob-Filho W., Otoch J.P., Pessoa A.F.M. (2023). Role of Nutritional Supplements on Gut-Muscle Axis Across Age: A Mini-Review. Cell Physiol. Biochem..

[66] Chou M.Y., Wong Y.C., Wang S.Y., Chi C.H., Wang T.H., Huang M.J., Huang P.H., Li P.H., Wang M.F. (2023). Potential Antidepressant Effects of a Dietary Supplement from Huáng Qí and its Complex in Aged Senescence-Accelerated Mouse Prone-8 mice. Front. Nutr..

[67] Lalonde R., Strazielle C. (2023). Probiotic Influences on Motor Skills: A Review. Curr. Neuropharmacol..

[68] Gupta N., El-Gawaad N.S.A., Mallasiy L.O., Gupta H., Yadav V.K., Alghamdi S., Qusty N.F. (2024). Microbial Dysbiosis and the Aging Process: A Review on the Potential Age-Deceleration Role of *Lactiplantibacillus plantarum*. Front. Microbiol..

[69] Giron M., Thomas M., Dardevet D., Chassard C., Savary-Auzeloux I. (2022). Gut Microbes and Muscle Function: Can Probiotics Make Our Muscles Stronger?. J. Cachexia Sarcopenia Muscle.

[70] Walsh M.E., Bhattacharya A., Sataranatarajan K., Qaisar R., Sloane L., Rahman M.M., Kinter M., Van Remmen H. (2015). The Histone Deacetylase Inhibitor Butyrate Improves Metabolism and Reduces Muscle Atrophy during Aging. Aging Cell.

[71] de la Motte S.J., Gribbin T.C., Lisman P., Murphy K., Deuster P.A. (2017). Systematic Review of the Association between Physical Fitness and Musculoskeletal Injury Risk: Part 2-Muscular Endurance and Muscular Strength. J. Strength. Cond. Res..

[72] Choi H.S., Jang Y.-J., Oh I., Chung J.H., Moon J.S. (2024). *Limosilactobacillus reuteri* ID-D01 Improves Exercise Performance and Reduces Muscle Fatigue in C57BL/6 mice through Regulation of Oxidative Capacity. J. Funct. Foods.

[73] Yu C.H., Lai C.C., Chen J.H., Chen I.C., Tai H.L., Fu S.K. (2023). Effect of *Lactobacillus plantarum* PS128 on Neuromuscular Efficiency After a Half-Marathon. Front. Physiol..

[74] Maruta H., Yoshimura Y., Araki A., Kimoto M., Takahashi Y., Yamashita H. (2016). Activation of AMP-Activated Protein Kinase and Stimulation of Energy Metabolism by Acetic Acid in L6 Myotube Cells. PLoS ONE.

[75] Mach N., Fuster-Botella D. (2017). Endurance Exercise and Gut Microbiota: A review. J. Sport Health Sci..

[76] Lefevre C., Bindels L.B. (2022). Role of the Gut Microbiome in Skeletal Muscle Physiology and Pathophysiology. Curr. Osteoporos. Rep..

[77] Roberts J.D., Suckling C.A., Peedle G.Y., Murphy J.A., Dawkins T.G., Roberts M.G. (2016). An Exploratory Investigation of Endotoxin Levels in Novice Long Distance Triathletes, and the Effects of a Multi-Strain Probiotic/Prebiotic, Antioxidant Intervention. Nutrients.

[78] Haywood B.A., Black K.E., Baker D., McGarvey J., Healey P., Brown R.C. (2014). Probiotic Supplementation Reduces the Duration and Incidence of Infections but not Severity in Elite Rugby Union Players. J. Sci. Med. Sport.

[79] Coleman J.L., Hatch-McChesney A., Small S.D., Allen J.T., Sullo E., Agans R.T., Fagnant H.S., Bukhari A.S., Karl J.P. (2022). Orally Ingested Probiotics, Prebiotics, and Synbiotics as Countermeasures for Respiratory Tract Infections in Nonelderly Adults: A Systematic Review and Meta-Analysis. Adv. Nutr..

[80] Peluso M.A., Guerra de Andrade L.H. (2005). Physical Activity and Mental Health: The Association between Exercise and Mood. Clinics.

[81] Mohr A.E., Pyne D.B., Leite G.S.F., Akins D., Pugh J. (2024). A Systematic Scoping Review of Study Methodology for Randomized Controlled Trials Investigating Probiotics in Athletic and Physically Active Populations. J. Sport Health Sci..

[82] Bressa C., Bailén-Andrino M., Pérez-Santiago J., González-Soltero R., Pérez M., Montalvo-Lominchar M.G., Maté-Muñoz J.L., Domínguez R., Moreno D., Larrosa M. (2017). Differences in Gut Microbiota Profile between Women with Active Lifestyle and Sedentary Women. PLoS ONE.

[83] Williams C.J., Torquati L., Li Z., Lea R.A., Croci I., Keating E., Little J.P., Eynon N., Coombes J.S. (2022). Oligofructose-Enriched Inulin Intake, Gut Microbiome Characteristics, and the VO_2_ Peak Response to High-Intensity Interval Training in Healthy Inactive Adults. J. Nutr..

[84] Zhou Y., Chu Z., Luo Y., Yang F., Cao F., Luo F., Lin Q. (2023). Dietary Polysaccharides Exert Anti-Fatigue Functions via the Gut-Muscle Axis: Advances and Prospectives. Foods..

[85] Jayachandran M., Chen J., Chung S.S.M., Xu B. (2018). A Critical Review on the Impacts of β-glucans on Gut Microbiota and Human Health. J. Nutr. Biochem..

[86] Zhang N., Mao X., Li R.W., Hou E., Wang Y., Xue C., Tang Q. (2017). Neoagarotetraose Protects Mice against Intense Exercise-Induced Fatigue Damage by Modulating Gut Microbial Composition and Function. Mol. Nutr. Food Res..

[87] Berg D., Clemente J.C., Colombel J.F. (2015). Can Inflammatory Bowel Disease be Permanently Treated with Short-Term Interventions on the Microbiome?. Expert. Rev. Gastroenterol. Hepatol..

[88] Jeukendrup A.E. (2004). Carbohydrate Intake during Exercise and Performance. Nutrition.

[89] Hanachi M., Manichanh C., Schoenenberger A., Pascal V., Levenez F., Cournède N., Doré J., Melchior J.C. (2019). Altered Host-Gut Microbes Symbiosis in Severely Malnourished Anorexia Nervosa (AN) Patients Undergoing Enteral Nutrition: An Explicative Factor of Functional Intestinal Disorders?. Clin. Nutr..

[90] Zhao L., Zhang Q., Ma W., Tian F., Shen H., Zhou M. (2017). A Combination of Quercetin and Resveratrol Reduces Obesity in High-Fat Diet-Fed Rats by Modulation of Gut Microbiota. Food Funct..

[91] van Wijck K., Wijnands K.A., Meesters D.M., Boonen B., van Loon L.J., Buurman W.A., Dejong C.H., Lenaerts K., Poeze M. (2014). *L-citrulline* Improves Splanchnic Perfusion and Reduces Gut Injury during Exercise. Med. Sci. Sports Exerc..

[92] Chunduri A., Reddy S.D.M., Jahanavi M., Reddy C.N. (2022). Gut-Brain Axis, Neurodegeneration and Mental Health: A Personalized Medicine Perspective. Indian. J. Microbiol..

[93] Montagnani M., Bottalico L., Potenza M.A., Charitos I.A., Topi S., Colella M., Santacroce L. (2023). The Crosstalk between Gut Microbiota and Nervous System: A Bidirectional Interaction between Microorganisms and Metabolome. Int. J. Mol. Sci..

[94] Mittal R., Debs L.H., Patel A.P., Nguyen D., Patel K., O’Connor G., Grati M., Mittal J., Yan D., Eshraghi A.A. (2017). Neurotransmitters: The Critical Modulators Regulating Gut-Brain Axis. J. Cell. Physiol..

[95] Bravo J.A., Forsythe P., Chew M.V., Escaravage E., Savignac H.M., Dinan T.G., Bienenstock J., Cryan J.F. (2011). Ingestion of *Lactobacillus* Strain Regulates Emotional Behavior and Central GABA Receptor Expression in a Mouse via the Vagus Nerve. Proc. Natl. Acad. Sci. USA.

[96] Choi J., Kim Y.K., Han P.L. (2019). Extracellular Vesicles Derived from *Lactobacillus plantarum* Increase BDNF Expression in Cultured Hippocampal Neurons and Produce Antidepressant-like Effects in Mice. Exp. Neurobiol..

[97] Ranuh R., Athiyyah A.F., Darma A., Risky V.P., Riawan W., Surono I.S., Sudarmo S.M. (2019). Effect of the Probiotic *Lactobacillus plantarum* IS-10506 on BDNF and 5HT stimulation: Role of Intestinal Microbiota on the Gut-Brain Axis. Iran. J. Microbiol..

[98] Gouttebarge V., Castaldelli-Maia J.M., Gorczynski P., Hainline B., Hitchcock M.E., Kerkhoffs G.M., Rice S.M., Reardon C.L. (2019). Occurrence of Mental Health Symptoms and Disorders in Current and Former Elite Athletes: A Systematic Review and Meta-Analysis. Br. J. Sports Med..

[99] Herzig S., Shaw R.J. (2018). AMPK: Guardian of Metabolism and Mitochondrial Homeostasis. Nat. Rev. Mol. Cell Biol..

[100] Lin S.C., Hardie D.G. (2018). AMPK: Sensing Glucose as well as Cellular Energy Status. Cell Metab..

[101] Janzen N.R., Whitfield J., Hoffman N.J. (2018). Interactive Roles for AMPK and Glycogen from Cellular Energy Sensing to Exercise Metabolism. Int. J. Mol. Sci..

[102] Tsuji A., Yoshikawa S., Ikeda Y., Taniguchi K., Sawamura H., Morikawa S., Nakashima M., Asai T., Matsuda S. (2023). Tactics with Prebiotics for the Treatment of Metabolic Dysfunction-Associated Fatty Liver Disease via the Improvement of Mitophagy. Int. J. Mol. Sci..

[103] Lew L.C., Hor Y.Y., Jaafar M.H., Lau A.S.Y., Ong J.S., Chuah L.O., Yap K.P., Azzam G., Azlan A., Liong M.T. (2019). *Lactobacilli* modulated AMPK Activity and Prevented Telomere Shortening in Ageing Rats. Benef. Microbes.

[104] Jeong H.W., Cho S.Y., Kim S., Shin E.S., Kim J.M., Song M.J., Park P.J., Sohn J.H., Park H., Seo D.B. (2012). Chitooligosaccharide Induces Mitochondrial Biogenesis and Increases Exercise Endurance through the Activation of Sirt1 and AMPK in rats. PLoS ONE.

[105] Nataraj B.H., Ali S.A., Behare P.V., Yadav H. (2020). Postbiotics-parabiotics: The New Horizons in Microbial Biotherapy and Functional Foods. Microb. Cell Fact..

[106] Carey R.A., Montag D. (2021). Exploring the Relationship between Gut Microbiota and Exercise: Short-Chain Fatty Acids and their Role in Metabolism. BMJ Open Sport Exerc. Med..

[107] den Besten G., van Eunen K., Groen A.K., Venema K., Reijngoud D.J., Bakker B.M. (2013). The role of Short-Chain Fatty Acids in the Interplay between Diet, Gut Microbiota, and Host Energy Metabolism. J. Lipid Res..

[108] Kim S.H., Huh C.S., Choi I.D., Jeong J.W., Ku H.K., Ra J.H., Kim T.Y., Kim G.B., Sim J.H., Ahn Y.T. (2014). The Anti-Diabetic Activity of *Bifidobacterium lactis* HY8101 In Vitro and In Vivo. J. Appl. Microbiol..

[109] Nan X., Zhao W., Liu W.H., Li Y., Li N., Hong Y., Cui J., Shang X., Feng H., Hung W.L. (2023). *Bifidobacterium animalis* subsp. *lactis* BL-99 Ameliorates Colitis-Related Lung Injury in Mice by Modulating Short-Chain Fatty Acid Production and Inflammatory Monocytes/Macrophages. Food Funct..

[110] Peng Y., Zhao L., Hu K., Yang Y., Ma J., Zhai Y., Jiang Y., Zhang D. (2022). Anti-Fatigue Effects of *Lycium barbarum* Polysaccharide and Effervescent Tablets by Regulating Oxidative Stress and Energy Metabolism in Rats. Int. J. Mol. Sci..

[111] Sies H. (2015). Oxidative Stress: A Concept in Redox Biology and Medicine. Redox Biol..

[112] Reardon T.F., Allen D.G. (2009). Iron Injections in Mice Increase Skeletal Muscle Iron Content, Induce Oxidative Stress and Reduce Exercise Performance. Exp. Physiol..

[113] Lee M.C., Ho C.S., Hsu Y.J., Huang C.C. (2022). Live and Heat-Killed Probiotic *Lactobacillus paracasei* PS23 Accelerated the Improvement and Recovery of Strength and Damage Biomarkers after Exercise-Induced Muscle Damage. Nutrients.

[114] Chen L.H., Huang S.Y., Huang K.C., Hsu C.C., Yang K.C., Li L.A., Chan C.H., Huang H.Y. (2019). *Lactobacillus paracasei* PS23 Decelerated Age-related Muscle Loss by Ensuring Mitochondrial Function in SAMP8 Mice. Aging.

[115] Zhang J., Chen L., Zhang L., Chen Q., Tan F., Zhao X. (2021). Effect of *Lactobacillus fermentum* HFY03 on the Antifatigue and Antioxidation Ability of Running Exhausted Mice. Oxid. Med. Cell Longev..

[116] Hu S., Kuwabara R., de Haan B.J., Smink A.M., de Vos P. (2020). Acetate and Butyrate Improve β-cell Metabolism and Mitochondrial Respiration under Oxidative Stress. Int. J. Mol. Sci..

[117] Zhonghui Z., Xiaowei Z., Fang F. (2014). *Ganoderma lucidum* Polysaccharides Supplementation Attenuates Exercise-Induced Oxidative Stress in Skeletal Muscle of Mice. Saudi J. Biol. Sci..

[118] Hu X., Mu L., Zhu L., Chang X., Nie L., Wang L., Li G. (2021). *Lycium barbarum* Polysaccharides Attenuate Cardiovascular Oxidative Stress Injury by Enhancing the Keap1/Nrf2 Signaling Pathway in Exhaustive Exercise Rats. Mol. Med. Rep..

[119] Chen Z., Li S., Wang X., Zhang C.L. (2013). Protective effects of *Radix pseudostellariae* polysaccharides against exercise-induced oxidative stress in male rats. Exp. Ther. Med..

[120] Baird L., Yamamoto M. (2020). The Molecular Mechanisms Regulating the KEAP1-NRF2 Pathway. Mol. Cell Biol..

[121] Pedersen B.K., Bruunsgaard H., Jensen M., Krzywkowski K., Ostrowski K. (1999). Exercise and Immune Function: Effect of Ageing and Nutrition. Proc. Nutr. Soc..

[122] Simpson R.J., Campbell J.P., Gleeson M., Krüger K., Nieman D.C., Pyne D.B., Turner J.E., Walsh N.P. (2020). Can Exercise Affect Immune Function to Increase Susceptibility to Infection?. Exerc. Immunol. Rev..

[123] O’Brien M.T., O’Sullivan O., Claesson M.J., Cotter P.D. (2022). The Athlete Gut Microbiome and its Relevance to Health and Performance: A Review. Sports Med..

[124] Gusmao-Silva G., Aguiar S.L.F., Miranda M.C.G., Guimarães M.A., Alves J.L., Vieira A.T., Cara D.C., Miyoshi A., Azevedo V.A., Oliveira R.P. (2020). Hsp65-Producing *Lactococcocus lactis* Prevents Antigen-Induced Arthritis in Mice. Front. Immunol..

[125] Deguine J., Barton G.M. (2014). MyD88: A Central Player in Innate Immune Signaling. F1000Prime Rep..

[126] Kon M., Ikeda T., Homma T., Akimoto T., Suzuki Y., Kawahara T. (2010). Effects of Acute Hypoxia on Metabolic and Hormonal Responses to Resistance Exercise. Med. Sci. Sports Exerc..

[127] Scherholz M.L., Schlesinger N., Androulakis I.P. (2019). Chronopharmacology of Glucocorticoids. Adv. Drug Deliv. Rev..

[128] Lane N.E. (2019). Glucocorticoid-Induced Osteoporosis: New Insights into the Pathophysiology and Treatments. Curr. Osteoporos. Rep..

[129] Wend P., Wend K., Krum S.A., Miranda-Carboni G.A. (2012). The Role of WNT10B in Physiology and Disease. Acta Physiol..

[130] Schepper J.D., Collins F., Rios-Arce N.D., Kang H.J., Schaefer L., Gardinier J.D., Raghuvanshi R., Quinn R.A., Britton R., Parameswaran N. (2020). Involvement of the Gut Microbiota and Barrier Function in Glucocorticoid-Induced Osteoporosis. J. Bone Min. Res..

[131] Chargo N.J., Schepper J.D., Rios-Arce N., Kang H.J., Gardinier J.D., Parameswaran N., McCabe L.R. (2023). *Lactobacillus reuteri* 6475 Prevents Bone Loss in a Clinically Relevant Oral Model of Glucocorticoid-Induced Osteoporosis in Male CD-1 Mice. JBMR Plus.

[132] Collins F.L., Irwin R., Bierhalter H., Schepper J., Britton R.A., Parameswaran N., McCabe L.R. (2016). *Lactobacillus reuteri* 6475 Increases Bone Density in Intact Females Only under an Inflammatory Setting. PLoS ONE.

[133] Sale C., Elliott-Sale K.J. (2019). Nutrition and Athlete Bone Health. Sports Med..

